# Influence of acid etching on internal bleaching with 16% carbamide peroxide

**DOI:** 10.1080/07853890.2021.1897351

**Published:** 2021-09-28

**Authors:** Ricardo Guerreiro, Inês Carpinteiro, Luís Proença, Mário Polido, Ana Azul

**Affiliations:** a Instituto Universitário Egas Moniz (IUEM), Egas Moniz Cooperativa de Ensino Superior, Caparica, Portugal; b Centro de Investigação Interdisciplinar Egas Moniz (CiiEM), Egas Moniz Cooperativa de Ensino Superior, Caparica, Portugal

## Abstract

**Introduction:**

Tooth bleaching has been an increasing demand [1] since tooth discolorations can have a negative impact on the social and psychological behaviour of patients. Internal bleaching (IB) can be used to treat teeth with endodontic treatment that are quite susceptible to pigmentation. The success of this technique depends on the ability to diffuse the active bleaching agent through pigments [2]. Thus, prior acid etching (PAE) of the pulp chamber has been proposed in order to remove the smear layer and to open the dentinal tubules, allowing a greater penetration of the bleaching agent and consequent increase in the treatment efficacy [2,3]. This pilot study was aimed to evaluate the effect of PAE, with 37.5% orthophosphoric acid, on *Commission Internationale de l’Éclairage* (CIE) L*a*b* parameters after IB with 16% carbamide peroxide (CP), as a function of application time.

**Materials and Methods:**

This study was approved by the Ethics Committee of Egas Moniz, CRL. Twenty sound molars were selected and randomly assigned to four experimental groups (*n* = 5, each): G1 – control group (without PAE or CP); G2 – 16% CP (without PAE); G3 – PAE for 15 *s* + 16% CP; G4 – PAE for 30 *s* + 16% CP. The initial colour was measured using a spectrophotometer and the correspondent CIE L*a*b* parameter values were obtained. Three sessions were performed every seven days. For the CG, a glycerine and carbopol placebo gel was applied. In the second and third sessions, for all groups the same procedures were performed except PAE step which was only applied in the first session of the respective groups. After 21 days, calcium hydroxide was placed on all teeth in order to neutralise the environment and standardise the experimental groups prior to restoration. The final colour was measured after 15 days, a restoration was performed with composite resin and the teeth were submitted to microtensile bond strength (μTBS) evaluation. For each fractured stick, the failure mode was classified according to its location [4]. Data was submitted to descriptive and comparative inferential statistical analysis. In the latter, a significance level of 5% was used.

**Results:**

A statistically significant increase in the mean L* parameter (luminosity) was observed after bleaching, with acid-etched groups showing the highest values. Mean a* and b* parameters (chroma) showed a statistically significant reduction in the groups submitted to PAE (G3, G4). Overall, these groups presented the lowest bond strength values. However, only G4 exhibited a statistically significant difference compared to G1 (*p*=.021) and to G2 (*p*=.010) while there was no statistically significant difference when considering G3 (*p*=.426).

**Discussion and conclusions:**

In light of the obtained results, the best clinic option will be to perform an orthophosphoric acid application for 15 s, prior to internal bleaching procedures, since tooth luminosity values increase and chroma values decrease, without affecting the microtensile bond strength. Thus, in this way, an improvement on internal bleaching outcome is achieved, without compromising the final restoration bond strength.
